# Supplementary material for: Exogenous MnSO4 Improves Productivity of Degenerated Volvariella volvacea by Regulating Antioxidant Activity
Source: J Fungi (Basel). 2024 Nov 27;10(12):825. doi: 10.3390/jof10120825 (PMC11678813; doi:10.3390/jof10120825)
Supplement: Supplementary file 1 [file jof-10-00825-s001.zip › jof-3279634-SI.pdf]

Supplementary Material

Supplementary Figure

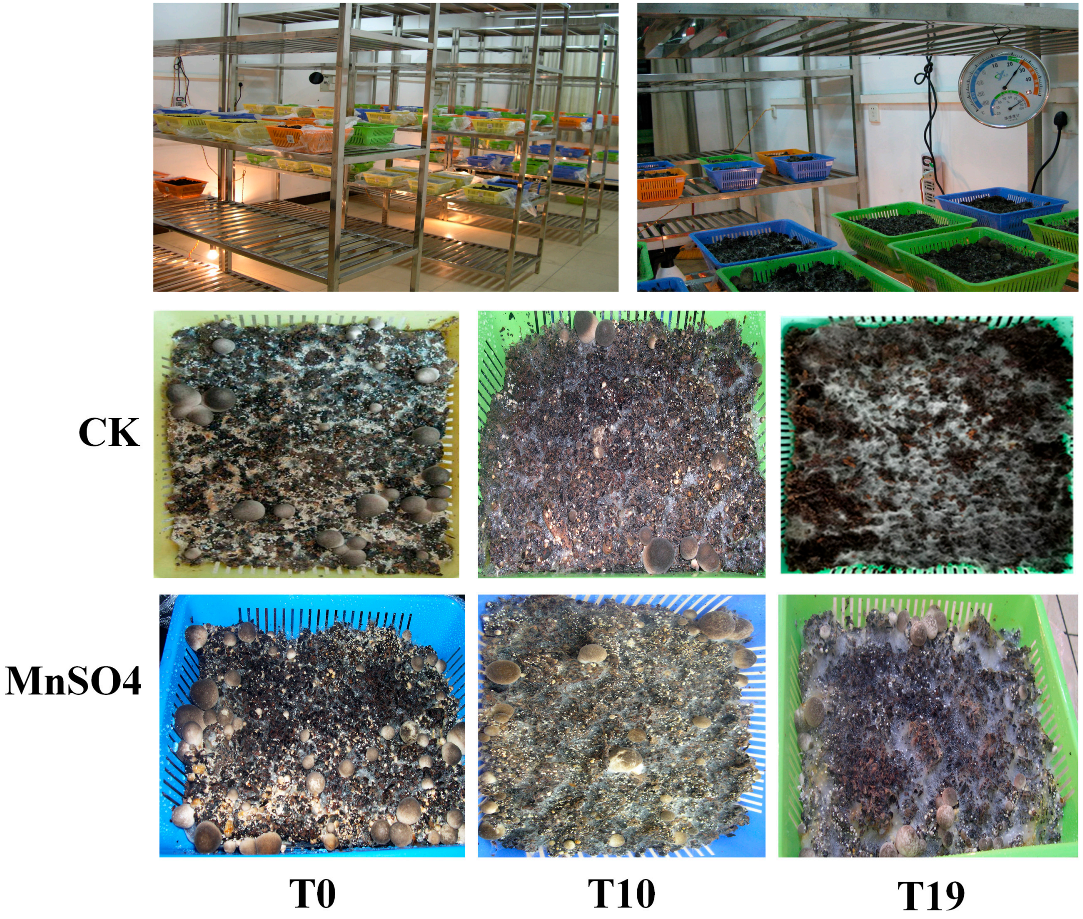

Figure S1: Effect of MnSO4 on *V. volvacea* cultivation test.
